# Domain-Specific Physical Activity and Sedentary Behavior in Older Adults With Mobility Disability Risk Residing in a Continuing Care Retirement Community: A Cross-Sectional Study

**DOI:** 10.1155/jare/7436862

**Published:** 2025-10-13

**Authors:** Jemimah O. Bakare, Soyoung Choi, Susan Aguiñaga, Ziyue Wang, Emerson Sebastião

**Affiliations:** ^1^Department of Health and Kinesiology, University of Illinois Urbana-Champaign, Urbana, Illinois, USA; ^2^College of Physical Education and Sports, Beijing Normal University, Haidian District, Beijing, China

**Keywords:** activity behavior, community dwelling facility, functional ability, senior living

## Abstract

**Introduction:**

This study investigated domain-specific physical activity (PA) and sedentary behavior (SB) among older adults living in a Continuing Care Retirement Community (CCRC) as a function of risk of mobility disability.

**Methods:**

Secondary cross-sectional data from 100 older CCRC residents were analyzed. The short physical performance battery (SPPB) assessed mobility disability risk, and PA and SB were self-reported. Data were analyzed using descriptive statistics, Mann–Whitney *U* test, and Quade's nonparametric ANOVA, with significance set at *p* < 0.05.

**Results:**

Fifty-nine participants had an SPPB score < 10, indicating high mobility disability risk, while 41 had a score ≥ 10, indicating low risk. The low-risk group had higher total PA (67.1 ± 41.8 vs. 49.2 ± 40.5, *p*=0.012) and leisure PA (30.5 ± 25.1 vs. 21.2 ± 23.5, *p*=0.035) minutes, and higher total sedentary minutes (645.8 ± 209.6 vs. 567.0 ± 290.8, *p*=0.007) and non–screen sedentary minutes (447.1 ± 182.7 vs. 350.0 ± 164.8, *p*=0.002) than the high risk group. After controlling for age, perceived health, and assistive device use, the differences between groups were no longer significant (*p* > 0.05).

**Discussion:**

Despite nonsignificant adjusted differences, our findings indicate overall low PA and high SB in the study participants. Given the well-documented benefits of PA, targeted interventions are needed to increase PA and reduce SB in this population.

## 1. Introduction

Older adults aged 65 and over currently constitute 16% of the U.S. population, and this figure is projected to rise to 22% by 2040 [1]. The aging population comes with individual, societal, and medical challenges, such as increasing prevalence of chronic diseases [2] and mobility disability [1]. Due to this demographic change and the significant impact of mobility on independent living and quality of life, mobility disability among older adults is a public health concern [3]. Mobility is a crucial component of healthy aging, defined as “*the process of developing and maintaining the functional ability that enables wellbeing in older age* [4].”

Mobility limitation, typically defined as difficulty walking a quarter mile or climbing a flight of stairs, is a common issue among older adults. It represents an early, potentially reversible stage of mobility loss and serves as a precursor to mobility disability, which is the inability to ambulate without significant difficulty or assistance [5]. Recent evidence suggests that the prevalence of mobility limitation among older adults has increased from 22.5% to nearly 47% [6, 7]. Additional studies have indicated that mobility limitations have been reported as prevalent in older adults affecting about 35% of those aged 70 years and most of those aged 85 years and over [6, 7]. This is concerning because mobility limitations have been associated with a wide range of adverse health outcomes, including but not limited to increased risk of falls, mobility disability, hospitalizations, mortality, reduced quality of life, and psychosocial problems [8–10]. There is further evidence suggesting that older adults with mobility limitations incur additional healthcare expenses, and more hospitalizations [9], and report low levels of physical activity (PA) [11].

PA is a key health behavior that can be assessed across four distinct domains: leisure-time (involves structured exercises and recreational activities), occupational (involves movements performed as part of individual's job), household/domestic (involves chores such as cleaning, gardening, and caregiving responsibilities), and transportation-related PA (includes walking or cycling for commuting purposes) [12]. Evaluating PA across these domains provides a comprehensive picture of one's movement patterns and overall activity levels, which are paramount for understanding its impact on health and well-being. Given the strong association between PA and health outcomes, the assessment of PA (across all domains) is particularly important for population with or at risk for mobility disability. To this end, PA has been highly recommended for older adults as a health promotion strategy to prevent or delay the onset of chronic diseases and conditions, and to maintain, restore, or improve physical function—including mobility [13]. For instance, a recent study observed that long-term participation in group PA program improves lower extremity muscle strength and delays age-related declines in walking speed and physical function in older adults, despite the effects of aging [14]. Despite the compelling evidence of the benefits of PA for the older population [13], regular PA engagement is limited among older adults [15], with additional evidence showing prevalent sedentary behavior (SB), in this segment of the population [16, 17]. Previous studies conducted in different housing and living arrangements for older adults, such as Continuing Care Retirement Communities (CCRCs)—a type of community-dwelling facility, have focused on older adults with different risk for mobility disability. These studies observed that older adults spent 79% of their awake time in sedentary time, 20% in low-intensity PA, and only 1% in moderate-to-vigorous PA; with few residents meeting the current PA guidelines [18, 19].

There is a paucity of studies exploring domain-specific PA and SB among older adults living in CCRCs. This is important because a significant number of older adults (i.e., nearly 819,000 residents; 70% women, 6% aged 65 and below, 13% aged 65–74 years; 31% aged 75–84 years, and 50% aged 85 years and above) currently live in assisted living facilities or CCRCs in the United States [20], and such facilities have different environment dynamics related to individual services (e.g., house cleaning and laundry services, meal preparation, onsite services such as mini-market, and hairdressing among others) that may limit PA participation in this population. In addition, mobility is an important ability within the healthy aging framework [21], and researchers have indicated the need for studies in the healthy aging framework specific to older adults presenting with mobility limitations or disability [22]. Aligned with this, two previous studies investigated the association between PA and/or the joint association between PA and sedentary time with mobility limitations in large samples of older adults [11, 23]. While these studies yield valuable insights, they did not specifically focus on older adults living in CCRCs. Additionally, both studies used accelerometers to assess PA and sedentary time. Although this provided an objective measurement, it did not allow for a qualitative evaluation of these behaviors, such as identifying specific domains of activity and SB. Thus, this study investigated domain-specific PA and SB among older adults living in a CCRC as a function of risk for mobility disability. We hypothesized that older adults with low risk for mobility disability would show higher levels of PA and less SB than those with high risk for mobility disability.

## 2. Materials and Methods

### 2.1. Study Design

This cross-sectional study conducted a secondary analysis using the data collected from a previous study that explored the patterns of SB, cognition, and physical function in older adults living in a CCRC [19, 24]. The original study was approved by a university Institution Review Board (Protocol# HS18-0011) and conducted in accordance with the Declaration of Helsinki. Additionally, all participants provided informed consent prior to data collection.

### 2.2. Participants and Setting

This study included data from older adults who were residents of a single CCRC facility located in the Midwest region of the United States. At the start of the data collection, the eligible population within the CCRC three eligible units comprised 236 individuals aged 60 and above. A priori power analysis was conducted using G^∗^ Power 3.1 to determine the appropriate sample size for comparing PA and SB between the high-risk and low-risk groups. Using an independent samples *t*-test (two-tailed), an expected moderate effect size (*d* = 0.5), an *α* of 0.05, and a statistical power of 0.80, the analysis indicated that a total sample of 128 participants (64 per group) was required to detect a significant difference. Due to funding constraints, data were collected from 100 participants. The referred CCRC offers comprehensive care and services for older adults living in different arrangements: independent apartments, duplex homes, personal care apartments, and licensed healthcare unit. The study specifically targeted residents from the first three living options who were fully or partially independent, purposefully excluding individuals from the licensed healthcare unit due to their need for continuous care. The referred facility served as both the recruitment site and the setting for the study, providing a familiar and controlled environment for the participants to complete all the assessments.

### 2.3. Measures

#### 2.3.1. Risk for Mobility Disability

The risk for mobility disability was determined based on the scores of the short physical performance battery (SPPB), which is a valid and reliable measure of lower-extremity function in older adults [25]. The internal consistent of the scale, assessed as Cronbach's alpha was 0.76 [25], and test–retest reliability was found to be 0.87 [26]. The SPPB comprises a three-part assessment that evaluates standing balance, gait speed, and lower extremity strength. Performance scores for each individual SPPB assessment and a total score aggregating the individual assessments are calculated as per standard SPPB protocol. Total SPPB scores range between 0 and 12, calculated by summing the standing balance, gait speed, and lower extremity strength categorical scores with higher scores reflecting better performance. Previous research demonstrated that SPPB scores are significantly associated with loss of ability to walk 400 m after 3 years [27]. For this study, the total SPPB score was used to categorize participants into two different groups: low and high risk for mobility disability adopting the cutoff of 10 points previously reported in the literature [27]. Forty-one participants had a score > 10 and were classified as low risk for future mobility disability (low-risk) and 59 had a score ≤ 10 and were classified as high risk for future mobility disability (high-risk).

#### 2.3.2. PA

PA was self-reported assessed using the Physical Activity Scale for the Elderly (PASE) [28], which is a valid (Caltrac sensor; *r* = 0.79; Cronbach's alpha = 0.69) and reliable (test–retest reliability = 0.75 [95% CI 0.69–0.80]) instrument to measure PA engagement in the older population [29]. The PASE is a 12-item instrument designed to assess PA in large samples of older adults over a 1-week period and includes activities commonly engaged in by older adults. This instrument uses frequency (recorded as never, seldom [1–2 days/week], sometimes [3–4 days/week], and often [5–7 days/week]), duration (categorized as less than 1 h, between 1 and 2 h, 2–4 h, or more than 4 h), and intensity level of activity participation across three life domains (i.e., recreational/leisure, housework, and work-related activities) over the previous week to assign a score, with higher scores indicating greater PA. Leisure activities include walking outside the home, light, moderate, strenuous sports and recreation, and muscle strengthening. Housework (light and heavy) includes lawn work/yard care, home repair, outdoor gardening, and caring for others (recorded as Yes/No). Paid or unpaid work, other than work that involves mostly sitting, is recorded in total hours per week.

#### 2.3.3. SB

SB was assessed using the Longitudinal Aging Study Amsterdam Sedentary Behavior Questionnaire (LASA-SBQ), a questionnaire specifically developed for the older adult population [30]. Scores from LASA-SBQ have been moderately (*r* = 0.35) associated with scores from accelerometer-derived sedentary data [30] and present with acceptable test–retest reliability (i.e., 0.71). The referred instrument includes 10 questions related to time spent in both screen and nonscreen sedentary time (NSST). Based on the composition of the questionnaire, three metrics were calculated and expressed as minutes per day: (a) NSST, (b) screen sedentary time (SST), and (c) total sedentary time (TST). NSST was calculated based on the sum of time spent in eight out of ten possible activities (i.e., napping, reading, listening to music, working, and hobby, talking with friends, transportation, and church). SST was calculated based on the sum of time spent in two out of ten activities: watching TV and computer. TST was calculated based on the sum of time spent in NSST and SST.

#### 2.3.4. Demographic and Health-Related Characteristics

For sample characterization purposes, a collection of demographics and health-related information was gathered using a questionnaire designed for the study. Demographic variables included age, sex, education, and ethnicity. Health variables included body mass index (BMI), use of mobility aid, number of diagnosed chronic diseases, number of prescribed and over-the-counter medication taken, and perceived health. Perceived health was assessed using a single question: “Would you say that, in general, your health is excellent, very good, good, fair, poor.” This was later recoded as excellent/very good, good, and fair/poor.

### 2.4. Procedures

All assessments were completed in a single session at the fitness center office and exercise space inside the CCRC facility. First, participants were asked to read and sign the informed consent, and a copy of the document was provided to them. After signing the informed consent, a trained research team member administered the questionnaires, which were completed in the following order for all participants: PA and SB, and demographic, health, and clinical questionnaires (Fitness Center office). The research team member was present in the room throughout the completion of the questionnaires to clarify or answer any question the participant may have. After the questionnaires, the participants were directed to the exercise space of the fitness center to complete the SPPB. Upon the completion of the physical function test, they received a modest cash compensation ($20) for their time. The data were collected from February 2018 to May 2018.

### 2.5. Statistical Analysis

The research team used SPSS Version 29 (IBM Corporation, Armory, NY) for the statistical analysis, with significance set at *p*  < 0 .05. The Kolmogorov–Smirnov test revealed that the PA and SB data were not normally distributed. Thus, descriptive statistics (median, 25th and 75th percentile) were used to display PA and SB information. However, for better comparison with previous studies conducted in different populations of older adults, we also reported its mean and standard deviation. Mann–Whitney *U* test was used to explore differences in PA and SB values between low risk and high risk. Quade's nonparametric analysis of covariance was used to compare differences between the two groups while controlling for age, perceived health, and use of an assistive device, using a hierarchical approach (Model 1: age; Model 2: age and perceived health; Model 3: age, perceived health, and use of an assistive device).

## 3. Results

Table 1 presents the sociodemographic and health characteristics of the participants (*n* = 100). One hundred percent of the participants were White. The mean age was 84.7 (SD:6.3), with those in the high-risk group (86.5; SD: 6.0 years) having a higher mean age than those in the low-risk group (82.1, SD: 5.9 years), *p* < 0.001. Approximately 70% of the participants were female, 60% reported attaining at least a college degree, and on average were classified as overweight. High blood pressure (61%), arthritis (59%), and high cholesterol (42%) were highly prevalent among participants. A significantly higher percentage of participants in the low-risk group (56.4%) reported their health as excellent/very good compared to the high-risk group (29.6%) (*p*=0.025). Additionally, a significantly higher percentage of participants in the high-risk group (39%) reported using assistive devices to walk compared to the low-risk group (2.4%) (*p* < 0.001).

Table 2 presents the mean, standard deviation, median and 25th and 75th percentile values for the PA and SB parameters, along with the associated nonparametric comparison test results. Briefly, the Mann–Whitney *U* tests indicated that the low-risk group reported significantly higher leisure time PA (30.5, SD: 25.1 vs. 21.2, SD: 23.5, *p*=0.035) and total PA (67.1, SD: 41.8 vs. 49.2, SD: 40.5, *p*=0.012) than the high-risk group, with no significant difference (*p* > 0.05) for the other domains (i.e., household and work-related PA). In terms of SB, the low-risk group showed significantly higher NSST (447.1, SD: 182.7 vs. 350.1, SD: 164.8, *p*=0.002) and total ST (645.8, SD: 209.6 vs. 567.1, SD: 290.8, *p*=0.007) compared to the high-risk group.

Table 3 displays in detail the results of the subsequent analysis of covariance using Quade's test, controlling for age, perceived health, and use of assistive device to walk. Briefly, significant differences between groups were observed only for NSST after controlling for age (Model 1; *p*=0.014), and after controlling for both age and perceived health (Model 2; *p*=0.045). The significance disappeared in model 3–becoming marginal—when NSST scores were adjusted for age, perceived health, and use of an assistive device (*p*=0.063). No significant group differences were observed for leisure PA (Mean [SE]: low-risk = 24.69 [4.07] vs. high-risk = 25.10 [3.37], *p*=0.309) or total PA (Mean [SE]: low-risk = 57.45 [6.94] vs. high-risk = 57.26 [5.75], *p*=0.534) after controlling for age, perceived health, and use of assistive device for walking (Model 3). The results also indicated no significant difference for total ST (Mean [SE]: low-risk = 636.77 [46.46] vs. high-risk = 586.20 [38.12], *p*=0.251).

## 4. Discussion

This study examined the differences in domain-specific PA and SB (i.e., sitting time—a proxy of SB) in older adults living in a CCRC based on their risk for mobility disability. Our main findings indicate that older adults with low risk for mobility disability reported significantly higher levels of total and leisure PA, and total and NSST than those with high risk for mobility disability. However, the differences were no longer significant after controlling for selected covariates.

Nearly 60% of the older adults in our sample presented with scores below 10 in the lower-extremity function test, which point toward some level of mobility limitation—a potentially reversible stage of mobility loss and were classified as high risk for mobility disability [27]. Interestingly, a Canadian survey indicated that mobility disability is highly prevalent across all age groups for females, with the largest gap observed among those aged 65 years and over with 22.5% of females reporting mobility disability compared to 18.3% of males [31]. Our results show nearly twice the prevalence of mobility limitation (59%) compared to the findings from the China Health and Retirement Longitudinal Study, which reported a standardized prevalence of 30.4% among older adults living in the community [32]. Such discrepancies could be partially explained by the origin of the sample (CCRC versus older adults from the community) and the difference in the assessment of mobility limitation.

Our findings suggest that older adults classified as low risk for mobility disability reported significantly higher total and leisure PA, as well as total and NSST, compared to the high-risk group. However, no significant differences were observed between groups for household and work-related PA or SST. These results corroborate the findings of a previous study that examined the association between accelerometer-derived PA and mobility limitation in a representative sample of 543 older adults. That study found that approximately 60% of older adults reporting mobility limitations had low objectively measured PA [11]. This difference could be partially explained by the fact that low-risk individuals are likely to have fewer chronic conditions and physical limitations (less used of assistive device), and better perception of overall health, allowing for greater participation in physical activities, particularly leisure activities. Previous research has consistently shown a strong association between multiple chronic conditions and mobility limitations [7], and in our study, low-risk older adults had fewer chronic diseases, and a lower prevalence of assistive device use compared to the high-risk group. In addition, a higher number of older adult in the low-risk group reported perceived their health as excellent/very good or good compared to the high-risk group (Table 1). Our findings further partially align with the existing literature, which suggests that older adults with lower risk of mobility disability are more likely to maintain an active lifestyle and experience less SB [23]. In our study, the low-risk group reported higher sedentary time compared to the high-risk group. Although complex, it is possible that older adults in the low-risk group—giving the better physical conditions, better overall health, and less chronic diseases—were more engaged in the activities offered by the facility, which could involve, for example, board games (an activity that classify as sedentary from the energy expenditure perspective). It is important to note that many participants in the high-risk group were already using assistive devices, indicating they may have already experienced mobility disability. The use of assistive devices can signal underlying mobility impairments that may limit their ability to engage in physical activities. This could partially explain the significant differences in PA levels between the low- and high-risk groups, as those in the high-risk group may already be dealing with the consequences of mobility limitations. However, after controlling for age, perceived health, and assistive device use, the analysis of covariance showed that older adults with low and high risk for mobility disability had similar levels of PA across all domains and sedentary time.

Despite the initial significant difference in PA observed between the two groups, it is important to highlight that both groups exhibited very low PA levels compared to older adults living in the community. One study conducted with 297 community-dwelling older adults reported PASE scores ranging from 110.5 (females aged 75–89 years) to 184.6 (males aged 65–74 years) [33]. Another study observed an overall PASE score of 152.6 for community-dwelling older adults and 11.8 for those living in assisted living [34]. Additionally, data from the Canadian Longitudinal Study on Aging, which included 36,701 community-dwelling women and men aged 45–85 years free of mobility limitations, demonstrated that PASE scores vary with age. For example, among older females (75–80 years), PASE scores for the 5th, 50th, and 95th percentiles were 40, 90–110, and 180–200, respectively. Among males in the same age group, the corresponding scores were 50, 110–120, and 200–220 [35]. Although we did not analyze our data according to sex, 70% of our participants were female. The overall PASE score in our study was approximately 56, with the low-risk group reporting a score of 67 and the high-risk group 49. Collectively, these findings suggest that the PASE scores of our participants are comparable to those in the 5th percentile of older adults living in the community, indicating that those living in CCRCs may be at higher risk for physical inactivity. This underscores the need for CCRCs to implement more infusive PA programming and provide greater opportunities for residents to be physically active.

Researchers have suggested that about 30% of older adults (range of 22.5%–46.7% in various studies) have mobility limitations [36]. Understanding the patterns of PA and SB in older adults living in CCRC communities are critical due to the strong associations with individual's overall health and mobility [37], and the different environmental dynamics, mostly related to amenities (i.e., features and services provided) that may limit PA participation compared to the general community. This may be further important for the development and delivery of strategies and intervention in this environment. Previous work underscored that many older adults with mobility limitations are inactive and do not have the knowledge and skills to increase PA [38]. By contrast, PA can improve mobility in older adults with mobility limitations [39], and it has multiple positive effects on the aging process [40, 41]. Despite the lack of significant difference between groups (i.e., low- vs. high-risk) on PA and SB, the values for PA and SB can be considered low and high, respectively. Studies have shown that physical inactivity and SB are each independent determinants of adverse health [42, 43]. Low PA and high SB present a global health challenge, and they are particularly important in older adults—especially those living in CCRC facilities—as PA declines and SB increases with increasing age.

To the best of our knowledge, this is the first study conducted to examine domain-specific PA and SB in a CCRC, exploring potential differences in PA and SB as a function of mobility limitation status among older adults. This could be viewed as strength of the study. However, our findings should be interpreted with caution due to limitations. First, the homogeneity of the study sample (primarily female and one hundred percent White), and the relatively small sample size, which was not adequately powered, limit the generalizability of the findings to other older adult populations. For instance, the power analysis indicated that a total of 128 participants were necessary; however, only 100 older adults were assessed, which may have impacted the study's ability to detect significant differences. In addition, we did not use the extended PASE manual for additional activity examples. This may have impacted the results, as the majority of the sample consisted of older females, and some activities, such as fishing, are less common for this group. Additionally, the sample originated from a single CCRC in the Midwest, further restricting the applicability of the results. Furthermore, older adults residing in the referred CCRC but who did not participate in the study may have differed in PA, SB, or overall health, and a larger or more diverse sample with greater variability in PA and SB could potentially yield different insights into group differences. Second, the use of self-reported measures for PA and SB may have introduced recall bias. Third, the cross-sectional design of the study prevents any conclusions about cause-and-effect relationships. Future research should aim to adopt a longitudinal design, include a more diverse sample of older adults in terms of sociodemographic and geographical characteristics, and use a combined approach to assess PA and SB. This approach should combine qualitative tools (e.g., questionnaires) to explore PA behavior domains, along with objective measures (e.g., accelerometers and ActivPal) for more reliable information.

Our findings suggest that most older adults in our sample exhibited some degree of mobility limitation, classifying them as high risk for future mobility disability. Additionally, older adults at low risk for mobility disability were found to engage significantly more in leisure and total PA and reported higher total and NSST compared to those at high risk for mobility disability. However, after adjusting for age, perceived health, and the use of assistive devices, these differences were no longer significant. Mobility limitation is common among older adults and is a known risk factor for various adverse health outcomes, including but not limited to loss of independence, morbidity, mortality, and disability. As the global aging population continues to grow across the globe, along with the increasing number of older adults living in different types of senior housing, it is crucial to implement effective strategies to combat mobility limitations and disability. PA is associated with numerous positive outcomes, including a reduced risk of mobility limitations. Given the compelling benefits of PA, it is crucial to develop strategies and interventions to promote PA and reduce SB, particularly among those living in CCRCs and assisted living facilities.

## Figures and Tables

**Table 1:** General demographic and health characteristics of the sample.

	Overall (*n* = 100)	Low risk (*n* = 41)	High risk (*n* = 59)	*p* value
Age (years) mean (SD)	84.7 (6.3)	82.1 (5.9)	86.5 (6.0)	< 0.001
Sex, *n* (%)				
Female	70 (70)	29 (71)	41 (69)	0.89
Male	30 (30)	12 (29)	18 (31)	0.89
Education, *n* (%)				0.857
Less than college degree	40 (40.4)	17 (41.5)	23 (39.7)	
College degree or higher	59 (59.6)	24 (58.5)	35 (60.3)	
Perceived health, *n* (%)^∗^				0.025
Excellent/very good	38 (40.1)	22 (56.4)	16 (29.6)	
Good	41 (44.1)	14 (35.9)	27 (50.0)	
Fair/poor	14 (15.1)	3 (7.7)	11 (20.4)	
BMI (kg/m^2^)	27.2 (6.8)	27.7 (7.7)	27.0 (6.2)	0.61
Total medications, median (IQR)	6.0 (5.0)	6.0 (5.0)	6.0 (5.0)	0.20
Chronic diseases, *n* (%)-yes				
Arthritis	59 (59%)	20 (48.8%)	39 (66.1%)	0.065
Osteoporosis	27 (27%)	8 (19.5%)	19 (32.2%)	0.145
Type 2 diabetes	17 (17%)	4 (9.8%)	13 (22.0%)	0.100
High blood pressure	61 (61%)	21 (51.2%)	40 (67.8%)	0.074
High cholesterol	42 (42%)	21 (51.2%)	21 (35.6%)	0.137
Ischemic heart disease	9 (9%)	2 (4.9%)	7 (11.9%)	0.220
Heart failure	4 (4%)	2 (4.9%)	2 (3.4%)	0.722
Stroke	13 (13%)	3 (7.3%)	10 (16.9%)	0.150
Cancer	11 (11%)	8 (19.5%)	3 (5.1%)	0.025
Asthma/COPD	3 (3%)	2 (4.9%)	1 (1.7%)	0.367
Chronic pain	21 (21%)	9 (22.0%)	12 (20.3%)	0.880
Cataracts	36 (36%)	15 (36.6%)	21 (35.6%)	0.969
Glaucoma	7 (7%)	3 (7.3%)	4 (6.8%)	0.936
Anxiety	7 (7%)	6 (14.6%)	1 (1.7%)	0.014
Depression	7 (7%)	3 (7.3%)	4 (6.8%)	0.936
Nr. chronic diseases, mdn (IQR)	3.0 (3.0)	3.0 (2.0)	4.0 (3.0)	0.540
Assistive device to walk (Y/N)^¥^	24/70	1/38	23/32	< 0.001

*Note:* mdn: median, IQR: interquartile range.

Abbreviations: BMI, Body Mass Index; Y/N, Yes/No.

^¥^Missing data (*n* = 6).

^∗^Missing data (*n* = 7).

**Table 2:** Physical activity and sedentary behavior parameters overall and separated by risk for mobility disability of older adults.

	Overall (*n* = 100)		Low risk (*n* = 41)		High risk (*n* = 59)		*p* ^∗^
	Mean (SD)	Median (25th–75th)	Mean (SD)	Median (25th–75th)	Mean (SD)	Median (25th–75th)	
*Physical activity, score*							
Leisure	25.0 (24.5)	16.1 (11.8–31.1)	30.5 (25.1)	(11.8–45.3)	21.2 (23.5)	13.7 (8.6–21.5)	0.035
Household	23.2 (20.1)	25.0 (00.0–25.0)	26.6 (19.0)	(25.0–35.0)	20.8 (20.7)	25.0 (00.0–25.0)	0.097
Work	8.4 (17.4)	00.0 (00.0–12.0)	10.0 (20.5)	(00.0–14.2)	7.2 (14.9)	00.0 (00.0–9.0)	0.533
Total PA	56.6 (41.8)	43.2 (27.5–76.5)	67.1 (41.8)	(36.8–86.0)	49.2 (40.5)	37.5 (21.5–67.2)	0.012

*Sedentary time, min/day*							
NSST	390.2 (178.2)	360.0 (275.0–480.0)	447.1 (182.7)	435.0 (312.5–507.5)	350.1 (164.8)	310.0 (252.5–420.0)	0.002
SST	209.4 (148.5)	180.0 (135.0–270.0)	198.8 (89.1)	180.0 (127.5–262.5)	217.0 (179.5)	187.5 (135.0–270.0)	0.681
Total ST	599.7 (262.0)	540.0 (450.0–705.0)	645.8 (209.6)	615.0 (515.0–765.0)	567.1 (290.8)	510.0 (423.7–602.5)	0.007

*Note:* min/day: minutes per day.

Abbreviations: NSST, nonscreen sedentary time; PA, physical activity; SST, screen sedentary time; ST, sedentary time.

^∗^ *p* value for Mann–Whitney *U* test.

**Table 3:** Results of the Quade nonparametric test.

	F	DHF	DFE	*p* value
Model 1: Controlling for age				
Physical activity, score				
Leisure	2.128	1	97	0.148
Total PA	2.246	1	97	0.137
Sedentary time, min/day				
NSST	6.302	1	98	0.14
Total ST	3.590	1	98	0.061
Model 2: Controlling for age and perceived health				
Physical activity, score				
Leisure	1.187	1	97	0.279
Total PA	0.920	1	97	0.340
Sedentary time, min/day				
NSST	4.143	1	98	0.045
Total ST	2.238	1	98	0.138
Model 3: Controlling for age, perceived health, and use of assistive device to walk				
Physical activity, score				
Leisure	1.046	1	97	0.309
Total PA	0.389	1	97	0.534
Sedentary time, min/day				
NSST	3.534	1	98	0.063
Total ST	1.332	1	98	0.251

*Note:* DHF: degrees of freedom for hypothesis; min/day: minutes per day.

Abbreviations: DFE, degrees of freedom for error; NSST, nonscreen sedentary time; PA, physical activity; ST, sedentary time.

## Data Availability

The data that support the findings of this study are available upon reasonable request from the corresponding author. The data are not publicly available due to privacy or ethical restrictions.
